# Children’s Multisystem inflammatory syndrome with myopathy

**DOI:** 10.1590/0037-8682-0865-2020

**Published:** 2021-03-22

**Authors:** Zina Maria Almeida de Azevedo, Karla Gonçalves Camacho, Daniella Mancino da Luz Caixeta, Fernanda Lima-Setta, Tania Regina Dias Saad Salles, Fernanda Veiga de Góes, Marcio Fernandes Nehab, Carlos Eduardo da Silva Figueiredo, Maria de Fátima Monteiro Pereira Leite, Zilton Farias Meira de Vasconcelos, Juliana Gil Melgaço, Ana Paula Dinis Ano Bom, Daniella Campelo Batalha Cox Moore

**Affiliations:** 1 Fundação Oswaldo Cruz, Instituto Nacional Fernandes Figueira, Departamento de Pediatria, Unidade de Terapia Intensiva, Rio de Janeiro, RJ, Brasil.; 2 Universidade do Grande Rio, Faculdade de Medicina, Caxias, RJ, Brasil.; 3 Fundação Oswaldo Cruz, Instituto Nacional Fernandes Figueira, Departamento de Pediatria, Serviço de Neurologia, Rio de Janeiro, RJ, Brasil.; 4 Fundação Oswaldo Cruz, Instituto Nacional Fernandes Figueira, Departamento de Pediatria, Serviço de pediatria, Rio de Janeiro, RJ, Brasil.; 5 Fundação Oswaldo Cruz, Instituto Nacional Fernandes Figueira, Departamento de Pediatria, Serviço de Cardiopediatria, Rio de Janeiro, RJ, Brasil.; 6 Fundação Oswaldo Cruz, Instituto Nacional Fernandes Figueira, Laboratório de alta complexidade, Rio de Janeiro, RJ, Brasil.; 7 Fundação Oswaldo Cruz, Biomanguinhos, Laboratório de Tecnologia Imunológica, Rio de Janeiro, RJ, Brasil.; 8 Universidade Federal Fluminense, Departamento de Medicina Clínica, Niterói, RJ, Brasil.

**Keywords:** COVID-19, MIS-c multisystem inflammatory syndrome in children, Myositis

## Abstract

This report describes a case of multisystem inflammatory syndrome in a child that evolved with a pattern of toxic shock syndrome with coronary artery ectasia and neurological involvement, documented by magnetic resonance imaging, with changes in the corpus callosum and myopathy in the pelvic girdle and paravertebral musculature.

## INTRODUCTION

COVID-19 usually presents with respiratory symptoms and has a more severe course of evolution in adults. However, some reports with a more severe appearance in children have now emerged that usually begin with gastrointestinal symptoms^1^. One of the first reports was of an infant with COVID-19 developing Kawasaki disease (KD). In the United Kingdom, eight children with hyperinflammatory shock showed features similar to those of atypical KD, KD shock syndrome, and toxic shock syndrome^2^. In Bergamo, Italy, a study showed a thirty-fold higher incidence of KD during the pandemic^3^. Belhadjer et al. identified 35 children admitted to the pediatric intensive care unit (PICU) in France and Switzerland with cardiogenic shock, ventricular dysfunction, and severe inflammatory status^4^. Because of these cases, the WHO developed a definition of multisystem inflammatory syndrome (MIS-c), guiding online registration, to investigate the real incidence of this syndrome^5^. Since then, MIS-c has been identified in two studies in the USA, with 186^6^ and 570 patients^7^.

This report describes a case of MIS-c in a Brazilian child who started with clinical manifestations suggestive of acute abdomen and evidenced a neurological condition of hypotonia that slowly regressed without sequelae after hospital discharge.

## CASE REPORT

A seven-year-old male patient was admitted with daily fever, abdominal pain, and vomiting for seven days. He underwent videolaparoscopy on suspicion of appendicitis, but showed only mesenteric adenitis. He presented in the postoperative period with respiratory distress and hypoxemia on chest computed tomography (CT) showing pleural effusion and bilateral condensation without a ground-glass pattern. The patient was transferred to the PICU with respiratory failure (Glasgow 7) and underwent invasive mechanical ventilation. Systemic antibiotic therapy and vasopressors (norepinephrine and vasopressin) and dobutamine were started and later changed to adrenaline. He developed septic shock with refractory hypotension medicated with hydrocortisone 150 mg/m^2^/day. Laboratory findings showed leukocytosis of 14,800 cells/mm^3^ with neutrophilia and lymphopenia (880 cells/mm^3^), C-reactive protein (CRP) of 27.8 mg/dL, thrombocytopenia (35,000/mm^3^), hypoalbuminemia (2.13 g/dL), and a significant increase in aspartate aminotransferase (913 U/mL). 

**FIGURE 1:**
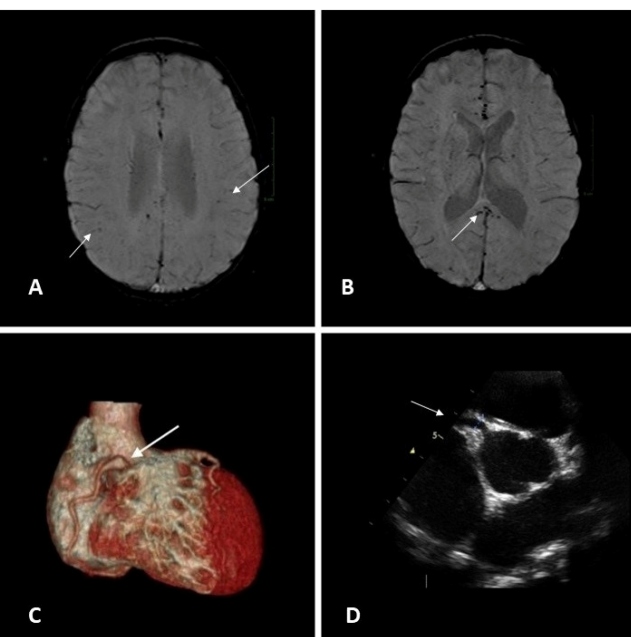
Brain resonance showing multiple areas of hypointense signal in the subcortical white matter of the cerebral hemispheres **(A)** and in the splenium of the corpus callosum **(B)**. The arrow in the coronary angiotomography points to the ectasia region of the right coronary **(C)**. Echocardiogram showing ectasia of the coronary artery **(D)**.

The diagnosis of MIS-c was suggested by the previous history of symptoms of sore throat, headache, and runny nose 30 days before admission; flu-like symptoms and anosmia in the parents in the same period; and the patient’s evolution. The patient also showed changes in inflammatory markers such as CRP (32.6 mg/dL), creatine phosphokinase (1,389 U/mL), ferritin (3,261.4 ng/mL), pro-calcitonin (31 ng/mL), IL-6 (194.63 pg/mL), and cardiac dysfunction markers such as NT-proBNP (11,055 pg/mL), CK-MB (28.36 ng/mL), and troponin I (0.96 ng/mL). The echocardiogram performed on the second day of hospitalization showed no involvement of the coronary arteries and had an ejection fraction of 73%. Immunoglobulin (2 g/kg/day) was administered given the severe condition and a suspected case of MIS-c. The reverse-transcriptase polymerase chain reaction (PCR-RT) was negative for SARS-CoV-2, but the IgG serology was positive. The patient recovered from septic shock within 24 hours with progressive weaning of vasoactive drugs that were finally suspended on the fourth hospitalization day. After 72 hours, despite clinical improvement, the patient had thrombocytopenia, international normalized ratio (INR) of 1.44, D-dimer level of 25,074 ng/mL, and CRP of 35 mg/dL. He was extubated on the fifth day of hospitalization. Thrombocytopenia resolved on the seventh day. The echocardiogram performed on the fifth hospitalization day when the patient was already extubated and clinically improved showed ectasia of the coronary artery, and the use of acetylsalicylic acid was started (Figure 1D). Coronary angiotomography on the eleventh hospitalization day confirmed a small ectasia of the right coronary artery and a tortuous left coronary artery (Figure 1C). On the ninth day, it was already possible to observe within normal values of CK-MB and troponin, but D-dimer still showed high values (4,000 ng/mL). After extubation, the patient was lucid, slightly lethargic, with generalized muscular hypotonia, overall reduced muscle strength, normal deep reflexes, normal anal and cremasteric reflexes, and no changes in the vibratory, tactile, or painful sensitivity tests. However, the patient demonstrated reduced proximal reflexes (tricipital, bicipital, and patellar) compatible with myositis. The cranial nerves remained unaltered. 

The diagnostic hypothesis of septic encephalopathy or neurological disorders associated with MIS-c was established. CSF analysis showed cellularity of only 5 cells/mm^3^ with 20% CD8+, with absence of IL-6 in the CSF. These findings suggested that neurological manifestations could be more related to indirect effects mediated by host CD8 + cytotoxic T cells than the effect of the cytokine storm. Brain magnetic resonance imaging (MRI) performed on the eleventh day of hospitalization showed multiple areas of hypointense signal suggestive of sparse hematic residue in the subcortical white matter of the cerebral hemispheres and notably in the splenium of the corpus callosum (Figure 1A and (1B). Muscle MRI showed multiple areas of signal alteration and contrast uptake in the pelvic girdle, lateral extensor, and minimal gluteus to the right and paravertebral musculature, suggesting myopathy, although the CPK level was already lower (67 U/mL). On this day, the patient was transferred to the nursing ward with progressive muscle tone improvement, and full recovery was observed 30 days after discharge from the PICU. This case report was approved by the Research Ethics Committee of the Fernandes Figueira Institute (IFF/FIOCRUZ).

## DISCUSSION

The patient met the WHO criteria for MIS-c^5^ and evidenced a neurological condition at a late stage where the inflammatory markers already showed improvement. The neurological condition observed with muscle weakness and radiological alteration in the corpus callosum seems to add more evidence to support the hypothesis of a virus-specific phenotype for the associated neurological syndrome suggested by a study in the United Kingdom that found similar neurological manifestations in four patients with MIS-c^8^. The mechanism involved still needs to be defined. Direct invasion of the central nervous system by SARS-CoV-2 is possible because the virus can access the central nervous system by entering the nasal mucosa, horizontal plate, and olfactory bulb retroactively via axonal transport^9^. Neurological manifestations may also be indirect effects mediated by host CD8+ cytotoxic T-cells, post-infectious immune responses with cross-reactivity, or vigorous inflammatory responses following a cytokine storm^9^. The reported patient did not show viral isolation in the CSF, which suggests an indirect lesion related to the immune response since the CSF cell pattern had 20% of TCD8+ cells, which is double the expected. Myopathy evidenced by MRI shows an intense muscular impairment that has not been described in pediatric patients with COVID-19. Considering that the patient only spent four days on mechanical ventilation with venous sedation without a muscle blocker, it is unlikely that we would relate this to the myopathy of the critically ill patient.

The elevation of cardiac enzymes and serum interleukin-6 (IL-6) levels reinforce one of the mechanisms proposed for myocardial injury, which is a cytokine storm triggered by an unbalanced immune response of proinflammatory and regulatory T-cells^10^. These two points have drawn our attention. First, there is a rapid decline in the levels of cardiac injury markers. A similar cardiac dysfunction condition has been described, suggesting that the mechanism of cardiac dysfunction in MIS-c is different from that in adults^6^. Second, although the patient did not show a characteristic phenotype of KD, the pattern of cardiac injury was similar to that observed in this disease that can affect the coronary arteries and whose main finding is myocardial edema without ischemic damage and with limited cell necrosis, as evidenced by mild to moderate elevations of troponin^4^. Evidence of exposure to SARS-CoV-2 by serology and not by PCR-RT was also described in a study that identified 570 cases of MIS-c in which the evidence of infection was obtained in 46% only by serology, which suggests that the syndrome may occur late as a consequence of the immune response^7^.

When MIS-C was first described, an article reflected the concern that if this syndrome was actually associated with SARS-CoV-2 infection as COVID-19 cases multiplied worldwide, it would not be difficult to associate a set of signs and symptoms with COVID-19^11^. The scientific evidence obtained as new cases were published shows that MIS-c is an uncommon manifestation (affects 2 out of every 100,000 people under 21 years of age) that typically occurs 2-4 weeks after infection with SARS-CoV-2, and the evidence of infection by PCR-RT is detected only in a small portion of patients^12^. Although the causal relationship is increasingly likely, it is essential to elucidate the pathophysiological mechanisms of MIS-c and clarify those manifestations that resemble KD, toxic shock syndrome, and macrophage activation syndrome.

We can highlight some important points regarding MIS-c: 1) it is an unusual pediatric manifestation; 2) it is one of the conditions where serology for SARS-CoV-2 provides more information in the diagnosis of active disease; 3) inflammatory markers such as C-reactive protein, D-dimer, troponin, and ferritin should be measured ; 4) cardiac dysfunction with coronary artery involvement may occur, so echocardiography is recommended; 5) gastrointestinal symptoms are important; 6) until the development of more specific protocols, corticosteroids and immunoglobulins must be considered; 7) it is necessary to exclude other diagnoses already known; 8) in cases with neurological manifestations, alteration of the corpus callosum should be investigated using MRI. 
